# Supplementary material for: Postmortem Changes in mRNA Expression and Tissue Morphology in Brain and Femoral Muscle Tissues of Rat
Source: Int J Mol Sci. 2025 Jul 22;26(15):7059. doi: 10.3390/ijms26157059 (PMC12346364; doi:10.3390/ijms26157059)
Supplement: Supplementary file 1 [file ijms-26-07059-s001.zip › ijms-3725605-supplementary.pdf]

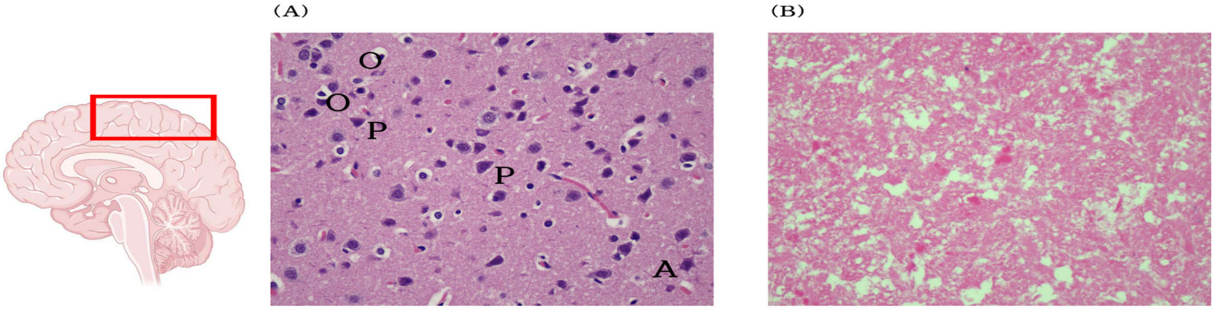

Supplementary Figure S1. Comparison of tissue composition of the brain cortex before (A) and after (B) histological changes (x400).

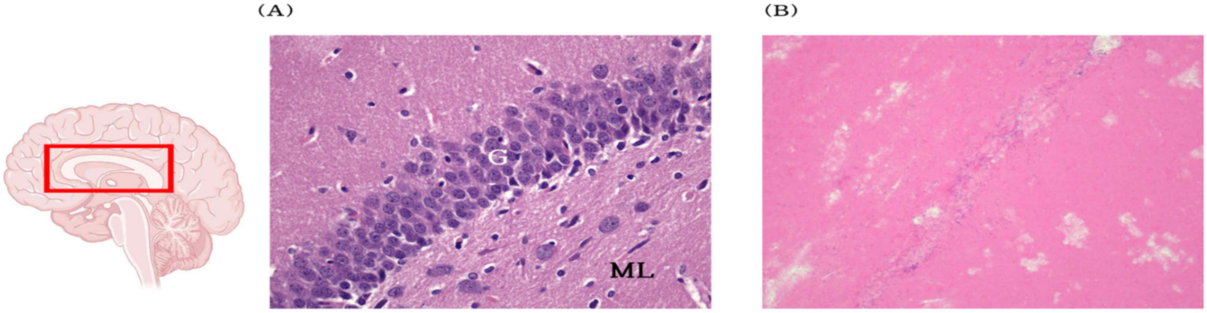

Supplementary Figure S2. Comparison of tissue composition of hippocampus of the brain before (A) and after (B) histological changes (x400).

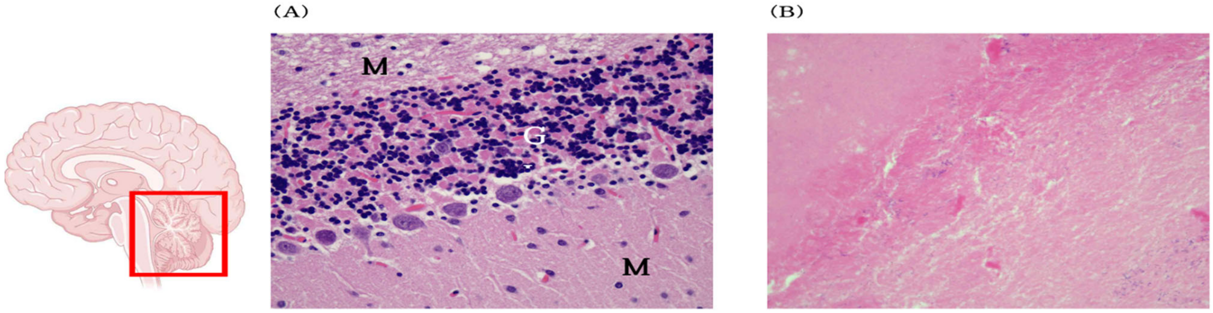

Supplementary Figure S3. Comparison of tissue composition of the cerebellum of the brain before (A) and after (B) histological changes (x400).

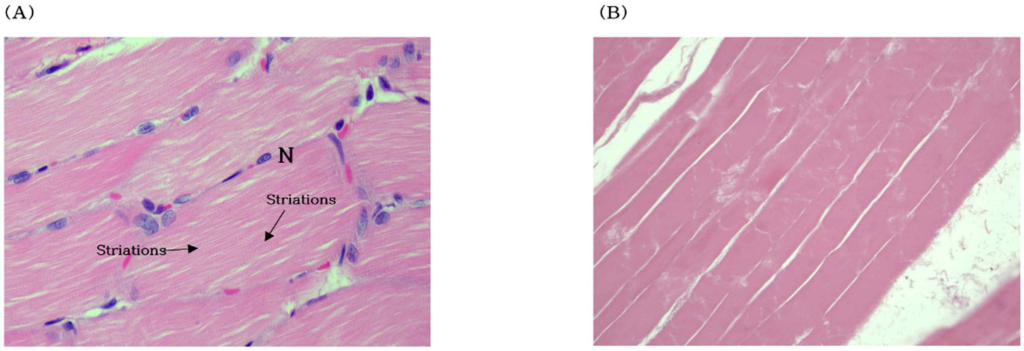

Supplementary Figure S4. Comparison of tissue composition of longitudinal sections of the femoral muscle before (A) and after (B) histological changes (x400).

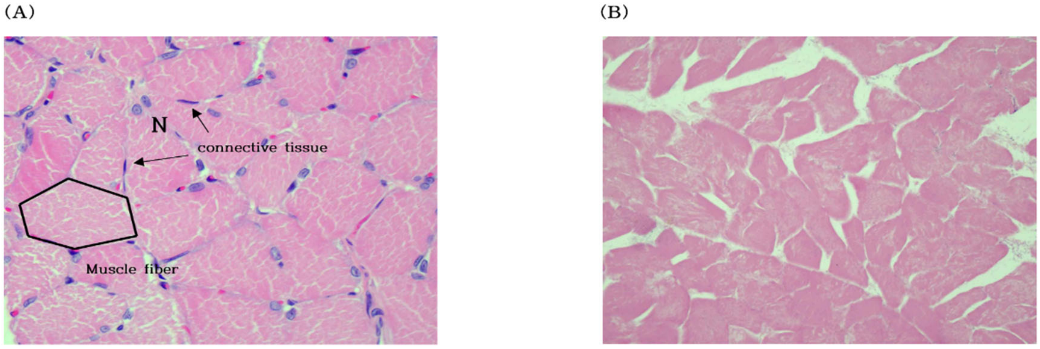

Supplementary Figure S5. Comparison of tissue composition of the transverse section in the femoral muscle before (A) and after (B) histological changes (x400).

Supplementary Table S1. Cycle threshold (Ct) values (mean ± SD and median) of 5S rRNA in brain and femoral muscle tissues at different postmortem intervals (PMIs) and storage temperatures (4 °C and 26 °C).

Temperature \ Time	Tissues	CT value	0h	6h	12h	24h	36h	48h	4d	6d	8d	10d	14d	21d
26°C	Brain	Mean ± SD	11.71 ± 1.03	11.24 ± 1.03	10.38 ± 0.36	10.76 ± 3.06	12.13 ± 1.97	17.29 ± 0.87	14.17 ± 0.89	14.44 ± 0.67	12.84 ± 0.76	11.89 ± 1.97	12.70 ± 2.08	11.81 ± 2.53
		Median	12.34	11.23	10.48	9.16	13.41	17.32	14.41	14.86	13.01	11.36	11.82	10.23
	Femoral muscle	Mean ± SD	10.46 ± 2.21	10.98 ± 2.64	10.20 ± 2.36	12.70 ± 0.84	11.24 ± 3.71	11.61 ± 2.61	11.81 ± 3.04	12.37 ± 2.87	12.55 ± 1.81	11.83 ± 2.47	15.20 ± 4.43	14.90 ± 3.82
		Median	10.12	11.43	10.05	12.87	11.25	12.55	13.08	11.77	13.08	11.76	13.92	15.14
4°C	Brain	Mean ± SD	12.40 ± 1.32	11.94 ± 1.49	9.88 ± 1.38	13.35 ± 2.00	13.31 ± 3.11	11.24 ± 1.52	13.81 ± 0.35	13.98 ± 0.80	15.20 ± 0.70	9.99 ± 0.70	11.63 ± 1.30	13.86 ± 4.91
		Median	13.32	11.27	9.68	13.51	13.99	12.17	13.97	13.72	15.65	10.37	11.01	11.47
	Femoral muscle	Mean ± SD	9.82 ± 2.78	10.61 ± 2.12	11.02 ± 2.22	12.11 ± 2.27	13.88 ± 2.43	14.00 ± 1.48	11.82 ± 3.03	11.53 ± 2.88	13.02 ± 1.91	13.60 ± 3.53	12.24 ± 2.30	13.37 ± 2.16
		Median	10.27	10.51	11.37	12.43	13.16	13.33	13.30	12.07	12.99	15.18	11.65	13.68

Supplementary Table S2. Cycle threshold (Ct) values (mean ± SD and median) of *Gapdh* in brain and femoral muscle tissues at different postmortem intervals (PMIs) and storage temperatures (4 °C and 26 °C).

**Temperature** \ **Time**	Tissues	CT value	0h	6h	12h	24h	36h	48h	4d	6d	8d	10d	14d	21d
**26°C**	Brain	Mean ± SD	21.62 ± 1.83	20.76 ± 0.81	21.03 ± 0.71	17.22 ± 5.25	21.65 ± 3.12	25.82 ± 0.88	24.17 ± 0.97	17.15 ± 2.18	20.70 ± 0.57	19.80 ± 1,47	25.61 ± 2.77	24.74 ± 3.69
		Median	22.61	20.27	21.12	16.3	23.24	26.29	24.39	16.16	20.86	20.31	23.65	22.4
	Femoral muscle	Mean ± SD	20.01 ± 1.85	19.94 ± 3.62	20.77 ± 3.32	17.84 ± 1,45	20.44 ± 2.62	19.66 ± 2,48	22.28 ± 2.12	21.48 ± 2.0.3	19.54 ± 1.35	21.73 ± 1.36	23.69 ± 1.52	21.29 ± 2.65
		Median	19.64	20.01	20.94	17.98	20.91	19.61	21.35	21.15	19.10	21.86	23.57	20.55
**4°C**	Brain	Mean ± SD	18.38 ± 0.76	19.73 ± 2.51	18.41 ± 2.62	21.76 ± 1.94	22.26 ± 2.67	20.08 ± 1.24	21.86 ± 0.88	22.84 ± 0.95	22.47 ± 2.55	20.17 ± 2.59	23.54 ± 2.69	22.24 ± 4.35
		Median	18.64	18.92	17.96	22.87	21.31	20.74	21.49	22.94	24.07	21.92	22.54	19.31
	Femoral muscle	Mean ± SD	18.25 ± 1.57	19.74 ± 2.10	18.90 ± 3.79	17.26 ± 1.68	22.79 ± 3.73	23.50 ± 2.63	19.92 ± 3.62	20.72 ± 3.57	19.94 ± 1.67	19.97 ± 3.86	20.33 ± 1.91	21.07 ± 2.15
		Median	18.41	19.09	17.34	19.28	22.06	24.19	20.20	21.80	20.09	21.76	20.33	21.98

Supplementary Table S3. Cycle threshold (Ct) values (mean ± SD and median) of *B2m* in brain and femoral muscle tissues at different postmortem intervals (PMIs) and storage temperatures (4 °C and 26 °C).

**Time** / **Temperature**	Tissues	CT value	0h	6h	12h	24h	36h	48h	4d	6d	8d	10d	14d	21d
**26℃**	Brain	Mean ± SD	32.94 ± 0.67	30.79 ± 1.92	31.39 ± 0.23	31.90 ± 1.62	34.72 ± 0.90	33.74 ± 0.17	32.51 ± 2.20	31.52 ± 3.43	32.47 ± 1.22	33.79 ± 2.68	32.64 ± 1.17	33.97 ± 1.03
		Median	33.22	31	31.35	32.51	34.1	33.66	32.56	33.58	31.77	35.66	32.05	33.99
	Femoral muscle	Mean ± SD	32.67 ± 0.80	34.11 ± 0.80	32.60 ± 0.68	32.52 ± 1.39	31.32 ± 1.27	33.94 ± 0.97	31.40 ± 0.66	33.63 ± 1.02	33.80 ± 1.07	33.38 ± 1,50	33.10 ± 1.11	33.29 ± 1.46
		Median	33	34	32.76	32.62	31.26	33.96	31.51	34	33.65	33.53	33.24	33.22
**4℃**	Brain	Mean ± SD	30.63 ± 3.58	29.49 ± 4.14	30.59 ± 3.67	31.93 ± 1.21	34.78 ± 1.37	32.11 ± 0.69	34.02 ± 1.07	33.84 ± 0.76	33.39 ± 0.47	29.42 ± 2.07	33.28 ± 1.63	33.51 ± 0.99
		Median	29.71	28.55	30.67	32.03	35.72	32.01	34.65	34.19	33.32	30.69	33.34	34.2
	Femoral muscle	Mean ± SD	32.54 ± 1.65	33.19 ± 1.30	33.09 ± 1.49	32.55 ± 1.31	33.42 ± 1.33	32.46 ± 0.90	33.85 ± 0.76	32.72 ± 0.65	33.85 ± 1.15	33.35 ± 1.49	33.39 ± 1.00	33.14 ± 1.53
		Median	32.37	33.58	33.25	33.35	33.37	32.36	33.91	32.68	34.35	33.77	33.72	33.29

Supplementary Table S4. Cycle threshold (Ct) values (mean ± SD and median) of *Sort1* in brain and femoral muscle tissues at different postmortem intervals (PMIs) and storage temperatures (4 °C and 26 °C).

Temperature \ Time	Tissues	CT value	0h	6h	12h	24h	36h	48h	4d	6d	8d	10d	14d	21d
**26°C**	Brain	Mean ± SD	22.70 ± 1.94	20.85 ± 1.19	24.42 ± 0.79	20.98 ± 5.17	25.59 ± 3.77	30.93 ± 1.95	28.59 ± 1.29	30.44 ± 0.75	32.74 ± 1.77	31.21 ± 1.39	30.26 ± 1.60	28.44 ± 2.23
		Median	21.39	21.68	24.69	19.39	27.81	31.22	28	29.95	33.97	31.29	29.21	27.13
	Femoral muscle	Mean ± SD	27.82 ± 1,59	27.04 ± 2.97	28.58 ± 2.91	27.36 ± 2.64	30.21 ± 2.68	29.82 ± 1.78	34.01 ± 1.62	30.48 ± 1.42	30.41 ± 0.89	30.32 ± 1.57	30.63 ± 1.54	29.77 ±1.77
		Median	27.98	27.53	29.35	27.45	30.91	29.77	34.29	30.14	30.60	30.41	30.92	29.52
**4°C**	Brain	Mean ± SD	23.77 ± 4.42	19.88 ± 3.44	21.38 ± 2.70	25.43 ± 1.68	27.85 ± 3.04	23.93 ± 1.84	32.58 ± 0.98	26.81 ± 1.68	26.01 ± 2.78	24.19 ± 3.93	27.76 ± 1.39	28.30 ± 3.06
		Median	26.83	18.72	21.49	26.36	27.01	24.24	31.93	27.91	27.66	25.86	28.72	29.63
	Femoral muscle	Mean ± SD	25.42 ± 1.67	27.38 ± 1.00	26.62 ± 2.16	29.42 ± 1.84	29.62 ± 2.14	31.40 ± 1.73	29.31 ± 3.90	29.11 ± 3.58	30.31 ± 2.05	28.39 ± 3.56	29.25 ± 1.07	29.63 ± 1.40
		Median	25.03	27.58	25.55	30.55	30.21	31.01	30.60	29.77	30.04	29.88	29.52	28.95

Supplementary Table S5. Relative gene expression (ΔCt values: mean ± SD and median) of target genes normalized to *Gapdh* in brain and femoral muscle tissues at various postmortem intervals (PMIs) under 4 °C and 26 °C.

Temperature \ Time	Tissues	△CT value	0h	6h	12h	24h	36h	48h	4d	6d	8d	10d	14d	21d
**26℃**	Brain	Mean ± SD	9.91 ± 2.47	9.93 ± 0.79	10.64 ± 0.71	6.45 ± 2.43	9.51 ± 1.18	8.53 ± 0.31	10.00 ± 0.09	2.71 ± 1.85	7.86 ± 0.87	7.91 ± 2.56	12.9 ± 0.87	12.93 ± 1.16
		Median	10.86	10.29	11.07	7.14	9.83	8.38	9.98	1.62	7.62	6.44	12.93	12.17
	Femoral muscle	Mean ± SD	12.03 ± 2.06	12.12 ± 3.77	12.97 ± 4.19	5.71 ± 2.16	7.63 ± 1.08	5.12 ± 2.41	6.63 ± 0.48	7.72 ± 1.12	4.58 ± 0.32	8.12 ± 1.46	5.12 ± 1.29	8.23 ± 7.87
		Median	11.34	14.42	14.08	5.99	7.19	6.35	6.39	7.10	4.5	7.81	4.9	3.12
**4℃**	Brain	Mean ± SD	5.98 ± 1.68	7.79 ± 1.04	8.53 ± 1.24	8.41 ± 0.71	8.95 ± 1.25	8.84 ± 0.29	8.05 ± 0.78	8.86 ± 1.62	7.28 ± 1.86	10.18 ± 1.90	11.91 ± 1.39	8.38 ± 0.87
		Median	5.84	7.65	8.28	8.20	9.16	8.71	8.16	9.78	8.34	11.32	11.53	7.84
	Femoral muscle	Mean ± SD	10.72± 0.67	12.20 ± 2.19	11.54 ± 3.92	9.58 ± 2.41	10.04 ± 0.68	7.18 ± 1.07	7.05 ± 0.44	8.37 ± 1.01	4.91 ± 0.78	7.47 ± 0.77	6.98 ± 1.06	7.37 ± 0.87
		Median	11.10	13.68	10.98	10.92	10.46	6.98	7.03	8.54	5.41	7.79	6.4	7.59

Supplementary Table S6. Relative gene expression (ΔCt values: mean ± SD and median) of target genes normalized to *B2m* in brain and femoral muscle tissues at various postmortem intervals (PMIs) under 4 °C and 26 °C.

Temperature \ Time	Tissues	△CT value	0h	6h	12h	24h	36h	48h	4d	6d	8d	10d	14d	21d
**26°C**	Brain	Mean ± SD	21.23 ± 0.43	19.55 ± 1.74	21.00 ± 0.34	21.24 ± 2.02	22.58 ± 1.77	16.45 ± 0.75	18.35 ± 2.11	17.08 ± 3.79	19.62 ± 1.96	21.91 ± 2.99	19.94 ± 1.09	22.16 ± 2.78
		Median	21.24	20.52	20.92	21.59	22.58	16.27	19.58	19.44	18.76	21.19	19.78	22.47
	Femoral muscle	Mean ± SD	24.39 ± 0.63	25.33 ± 1.32	25.18 ± 0.35	19.63 ± 1.77	16.93 ± 1.1	20.33 ± 0.76	16.6 ± 1.01	18.23 ± 0.6	20.09 ± 0.93	19.7 ± 1.57	14.59 ± 3.78	14.47 ± 2.97
		Median	24.3	25.73	25.18	18.75	16.2	20.73	17.18	18.01	20.29	18.68	12.42	14.12
**4°C**	Brain	Mean ± SD	18.23 ± 3.59	17.55 ± 5.21	20.71 ± 5.05	18.58 ± 2.81	21.46 ± 1.91	20.87 ± 1.65	20.21 ± 0.74	19.86 ± 0.84	18.20 ± 0.78	19.43 ± 1.39	21.66 ± 2.24	19.65 ± 4.67
		Median	19.17	18	20.99	19.86	21.78	20.83	20.52	19.49	18.27	20.09	20.25	20.65
	Femoral muscle	Mean ± SD	24.58 ± 2.08	25.48 ± 0.55	23.74 ± 2.26	23.32 ± 2.60	17.04 ± 1.18	17.23 ± 1.33	19.34 ± 1.11	18.49 ± 0.68	19.07 ± 1.98	17.41 ± 1.22	19.10 ± 1.42	17.29 ± 0.72
		Median	25.88	25.79	22.44	22.98	17.18	17.68	20.02	18.59	19.86	18.11	18.8	16.8

Supplementary Table S7. Relative gene expression (ΔCt values: mean ± SD and median) of target genes normalized to *Sort1* in brain and femoral muscle tissues at various postmortem intervals (PMIs) under 4 °C and 26 °C.

Temperature \ Time	Tissues	△CT value	0h	6h	12h	24h	36h	48h	4d	6d	8d	10d	14d	21d
**26℃**	Brain	Mean ± SD	10.99 ± 1.78	9.61 ± 1.57	14.04 ± 0.84	10.22 ± 2.21	13.46 ± 1.81	13.64 ± 2.81	14.42 ± 1.03	16.00 ± 0.65	19.90 ± 1.09	19.33 ± 1.96	17.56 ± 0.58	16.63 ± 0.49
		Median	11.13	9.18	14.45	10.23	14.4	13.9	15.02	16.37	20.34	19.69	17.39	16.37
	Femoral muscle	Mean ± SD	19.45 ± 1.22	18.94 ± 2.86	20.15 ± 3.45	16.21 ± 2.81	17.93 ± 1.24	15.82 ± 2.33	18.92 ± 1.53	15.93 ± 1.49	16.69 ± 0.29	14.69 ± 0.7	12.44 ± 2.61	10.8 ± 2.95
		Median	19.42	20.92	22.57	16.06	17.75	15.7	18.02	16.87	16.72	14.93	12.3	9.53
**4℃**	Brain	Mean ± SD	11.37 ± 5.20	7.94 ± 4.46	11.50 ± 1.34	12.08 ± 0.7	14.53 ± 1.07	12.69 ± 0.63	18.77 ± 1.33	12.83 ± 2.46	10.81 ± 2.09	14.20 ± 3.23	16.13 ± 1.15	14.43 ± 3.14
		Median	13.65	8.17	11.81	12.24	15.18	12.45	17.96	14.19	11.93	15.49	15.36	14.65
	Femoral muscle	Mean ± SD	18.05 ± 0.25	18.40 ± 0.86	18.89 ± 1.56	21.45 ± 0.96	16.24 ± 1.88	17.55 ± 0.81	18.46 ± 0.88	16.51 ± 0.67	17.12 ± 0.57	14.71 ± 0.95	15.81 ± 1.59	15.21 ± 2.20
		Median	17.97	18.47	18.99	21.44	17.56	17.47	18.49	16.6	17.74	15.36	16.44	16.29
